# Therapist-Reported Differences between Teletherapy via Phone and via Videoconferencing

**DOI:** 10.3390/brainsci13121714

**Published:** 2023-12-14

**Authors:** Vera Békés, Cédric Gill Ménard, Natale Schmitz, Katie Aafjes-van Doorn

**Affiliations:** 1 Ferkauf Graduate School of Psychology, Yeshiva University, 1165 Morris Park Ave, Bronx, NY 10461, USA; vera.bekes@yu.edu (V.B.); nschmitz@mail.yu.edu (N.S.); 2 Institut de Psychologie Contextuelle, Montreal, QC H2Y 2S9, Canada; cedric@contextpsy.com

**Keywords:** teletherapy, phone, videoconferencing, audio, attitudes, therapist, COVID-19

## Abstract

When therapists provide teletherapy they can choose between teletherapy via phone and teletherapy via videoconferencing, however, little is known about differences between using these two teletherapy formats. We aimed to compare therapists’ attitudes, level of professional self-doubt, and experience of the therapeutic relationship in sessions conducted via phone versus via videoconferencing. We administered an online survey to 117 therapists who had experience with sessions both via phone and videoconferencing. The results suggested that therapists’ attitudes and perceptions of the therapeutic relationship were similar. However, therapists’ level of professional self-doubt was higher in sessions conducted via videoconferencing. Therapists with previous experience of conducting teletherapy via phone perceived phone sessions more positively. Thus, familiarity with videoconferencing technology might be more important in shaping therapists’ attitudes and confidence than the type of technology per se. This highlights the importance of practicing with new technologies.

## 1. Introduction

### 1.1. The Use of Teletherapy

Teletherapy is a type of psychotherapy in which patient and therapist meet synchronously via audio (phone) or videoconferencing from different geographical locations [1,2,3]. Teletherapy treatments through phone and video can reduce mental health symptoms and are non-inferior to in-person therapy [4].

The use of teletherapy started in the 1960s, with phone therapy gaining popularity prior to video therapy [5]. Although most therapists had engaged in some clinical work via phone [6,7], they viewed teletherapy negatively. Most therapists much preferred in-person therapies [8,9,10]. After the sudden uptake of teletherapy during the pandemic, therapists started to be more acceptant of using teletherapy technology [11,12,13,14,15,16,17,18,19]. 

The Technology Acceptance Model (TAM) [20,21,22] provides a theoretical understanding of the process of technology uptake in different professions. Following the TAM model, therapists who have more experience with the teletherapy technology (i.e., those in the in-use acceptance phase of the TAM model) are more willing to engage with it [23,24]. Thus, as with any new technology, the recent teletherapy uptake has positively influenced therapists’ attitudes and their willingness to accept and engage with it.

This is important, because negative attitudes towards teletherapy have shown to be associated with higher levels of reported professional self-doubt, and with perceived lower quality of the therapeutic relationships [8,11,14,25]. Especially young therapists with little clinical experience, might experience professional self-doubt and might have concerns about the quality of the therapeutic relationship in teletherapy [12,26,27].

### 1.2. Teletherapy via Phone or Video

When therapists provide teletherapy they can choose between treatments via phone or via videoconferencing, or they can choose to use phone and video sessions in one treatment, however, making the choice is often difficult to make without research-based guidelines and factors to consider. Despite ample research on teletherapy acceptance more generally, no previous studies have examined therapists’ perspectives on the difference between teletherapy via phone versus via videoconferencing [28]. Although teletherapy treatments via phone and video appear to be equally effective [4], therapists might prefer one teletherapy format over the other. Some therapists have reported preferences for teletherapy via videoconferencing [29,30], possibly because of concerns regarding a lack of visual cues in phone sessions [8,31]. In contrast, other therapists have underlined the usefulness of teletherapy via the phone [31], because phones are easier to use and pose fewer technological challenges [16,26]. We posit that therapists’ perspectives on phone versus video sessions might depend on their attitudes towards these technologies, their professional self-doubt, and their perception of the quality of the therapeutic relationship in sessions conducted via phone and via videoconferencing.

### 1.3. Aims

In this study, we wanted to examine the differences between therapists’ perspectives on teletherapy via phone and teletherapy via videoconferencing. Specifically, we aimed to:

(1) Compare the various aspects of therapists’ attitudes towards the technology (i.e., therapy quality, ease of use, pressure and support to use it, convenience, and intention for using it in the future), their level of professional self-doubt, and their reported quality of the therapeutic relationship (working alliance and real relationship) in sessions conducted via phone and sessions conducted via videoconferencing;
(2) Establish whether differences between experiences in phone versus video sessions were moderated by therapists’ overall level of clinical experience, the number of sessions they presently conduct via phone/video, and their previous experience with providing teletherapy sessions via phone and video. Given the lack of previous studies comparing therapists’ experiences with different teletherapy formats, our study was explorative and we did not formulate specific hypotheses.

## 2. Methods

The present study is a secondary analysis of a large-scale dataset. The original study collected data on psychotherapists’ experiences and attitudes towards teletherapy after their transition from face-to-face to teletherapy during the pandemic (*n* = 1911). The study used a cross-sectional online survey design. The present study includes a subsample of 117 participants who practiced both phone and video therapy during the pandemic, and who completed all the relevant questionnaires for both treatment formats. Data were collected between 25 March and 29 June 2020.

### 2.1. Procedure

Therapists were recruited via professional email listservs, social media, and the authors personal and professional networks internationally, using convenience and snowball sampling methods. Licensed and trainee therapists were eligible to participate if they had at least seen one patient in remote sessions during the pandemic. Interested participants were led to an online platform, which provided additional information about the study. After providing consent, therapists were directed to an online, Qualtrics-based survey that included questions about professional experiences before and during the pandemic, as well as standardized self-report scales administered in a fixed order (see details in the Section 2), taking approximately 15 min to complete altogether. The study was approved by the institutional review board of Yeshiva University. Findings based on the original larger dataset have been previously published [11,13,32,33].

### 2.2. Measures

Each standardized measure was administered twice, first regarding video and then regarding phone therapy. Each measure’s instruction was adjusted accordingly to include either “in your video session” or “in your phone sessions”.

**Attitudes Toward Teletherapy Technology**. To assess the therapists’ attitudes toward remote therapy, we used the 21-item Attitudes Toward Online Therapy Questionnaire- Therapist version (UTAUT-T) [34]. For the aims of the current study, we used the original UTAUT-T to assess attitudes towards video sessions and used a modified version to assess attitudes towards video sessions. In the modified version we simply replaced the word “online” to “phone”. For example, the item “Working online is more convenient” was changed to “Working via the phone is more convenient” in the phone practice survey. The UTAUT-T is composed of six subscales: Therapy Quality Expectancy (9 items) to address perceptions of teletherapy quality, such as its effectiveness, ease of delivery, and general appeal; Ease of Use (4 items) to relate to the simplicity of teletherapy adoption, including possessing the required technical expertise, feeling confident, and comprehending technology usage; Pressure from Others (2 items), highlighting the opinions of significant individuals who believe the therapist should utilize teletherapy technology; Professional Support (2 items) to reflects the perceived support from organizations and colleagues specific to the therapist context; Convenience (2 items) to emphasize the time and cost-saving benefits teletherapy; and Behavioral Intention (2 items) to ask about intent and plan to use this format of practice after the pandemic. Respondents were asked to rate their level of agreement with each item on a 5-point Likert-type scale ranging from strongly disagree to strongly agree. Cronbach’s alpha in our study was .89 for phone and .90 for video sessions.

**Professional Self-doubt.** The Professional Self-Doubt Scale (PSD), a subset of the broader Development of Psychotherapists Common Core Questionnaire (DPCCQ) [35], was employed to evaluate the level of uncertainty therapists have concerning their own ability to help their patients [36]. This nine-item measure centers on a therapist’s self-perceived competence and confidence. Each item is rated on a 6-point Likert scale, ranging from 0 (never) to 5 (very often), culminating in a total score, with higher scores signifying a greater degree of self-doubt. The PSD has demonstrated acceptable internal consistency, with a Cronbach’s α of .77 [37]. The PSD was also administered twice, once for video and once for phone sessions. Chronbach’s alpha in our study was .85 for phone and .79 for video sessions.

**Real Relationship**. The Real Relationship Inventory Therapist Form (RRI-T) [38] was used to evaluate the authenticity and genuineness of the relationship between the patient and the therapist from the therapist’s perspective. Rooted in Gelso’s tripartite model, the RRI-T specifically captures the ongoing quality of this relationship, setting it apart from aspects of transference and the working alliance [38]. The RRI-T consists of 24 items, rated on a 5-point Likert scale ranging from “Strongly Disagree” (1) to “Strongly Agree” (5), with higher total scores indicating a more genuine and authentic relationship. While previous studies have established a notable correlation between the RRI-T and the Working Alliance Inventory (WAI) [39], particularly the WAI’s Bond subscale [40], ratings derived from the RRI-T have been observed to predict treatment progress and outcomes over and above the variance explained by the WAI [41,42]. Past research has affirmed the reliability of the RRI-T, with coefficient alphas typically ranging between 0.80 and 0.90 across different samples [43,44]. The RR-T was also administered twice, once for video and once for phone sessions. Chronbach’s alpha in our study was .85 for phone and .85 for video sessions.

**Working Alliance.** In the current study, the therapeutic alliance was assessed using the Working Alliance Inventory–Short Revised—Therapist version (WAI-SRT) [45], which is grounded in Bordin’s (1979) pantheoretical model of the working alliance [46]. This model encompasses three primary components: agreement on goals, agreement on tasks, and bond. The WAI-SRT comprises twelve items, with four positively worded items for each of the three subscales. Items are rated on a 5-point Likert scale, ranging from 1 (rarely) to 5 (always). The WAI-SRT has demonstrated adequate reliability and validity [45], and its scores have been associated with other alliance measures as well as the prediction of therapy outcomes [47,48]. To analyze the WAI-SRT data, the mean score was calculated, with higher scores representing a stronger working alliance between the therapist and patient. The WAI-SRT was also administered twice, once for video and once for phone sessions. Chronbach’s alpha in our study was .88 for phone and .97 for video sessions.

### 2.3. Data Analysis

Statistical analyses were completed using R Studio for Windows Version 2022.07.1 + 554 [49]. Preliminary data analysis to explore potential relationships between the study variables used zero-order Pearson’s correlations. To address the first research question regarding potential differences in attitudes towards phone and video teletherapy, professional self-doubt in phone versus video sessions, and the therapeutic relationship (working alliance and the real relationship), we used paired samples *t*-tests for the comparisons.

For investigating the second research question, that is, to test whether clinical experience, number of sessions presently conducted via phone/video, and previous experience with phone/video therapy moderate the differences between experiences with phone and video therapy, three-way mixed ANOVAs were performed. In the ANOVAs, the independent variable was phone/video and moderator variables were previous clinical experience, number of sessions presently conducted via phone/video, previous phone/video experience, and dependent variables in each analysis were attitudes, professional self-doubt, therapeutic alliance, and real relationship. Effect sizes were computed and reported as Cohen’s d for paired *t*-tests and partial eta-squared for ANOVAs. All tests were two-tailed, and the significance level was set at *p* < 0.05. Given the survey’s mandatory response method, there were no missing data in the dataset.

## 3. Results

### 3.1. Participants

In our study, most therapists were White (*n* = 90, 76.9%) and female (*n* = 85; 72.7%) with an average age of 52.72 (SD = 16.36). The vast majority were licensed (*n* = 110, 94%), and had some experience with either phone or video therapy before (*n* = 88, 75.2%, and *n* = 62, 53%, respectively); however, those with some experience typically only had one or two phone or video sessions per week previously. Nearly two thirds of therapists had more than 17 years of clinical experience (*n* = 75, 64.1%) and only a minority (*n* = 10; 8.6%) had 4 years or less of clinical experience. Detailed demographic data about the study sample are presented in Table 1.

When comparing the original sample and the subsample in the present study who used both phone and video sessions, the subsample was older, *t*(1909), *p* = .008, but did not differ significantly in other demographics and professional characteristics.

### 3.2. Preliminary Analysis

Older age, more clinical experience, and more previous experience with phone and video therapy were all associated with less professional self-doubt during phone and video sessions. For phone sessions, older age, more clinical experience, and more previous experience with phone and video therapy were also associated with more positive attitudes. However, for video sessions, attitudes towards video sessions were only related to previous experience with video but unrelated to age and clinical experience. Therapists with more patients in video sessions during the pandemic reported more positive attitudes towards video therapy, whereas number of patients in phone therapy was unrelated to attitudes or the therapeutic relationship in either phone or video therapy, see Table 2.

Furthermore, professional self-doubt on both phone and video was related to more negative attitudes towards phone and video therapy. More positive perceptions of the therapeutic relationship (working alliance and real relationship) were related to more positive attitudes towards both video and phone sessions, see Table 3.

### 3.3. Therapists’ Perceived Differences between Teletherapy via Phone and via Video

Therapists conveyed a positive attitude towards conducting psychotherapy via video and phone, with no significant difference between the two teletherapy formats. Therapists also did not report significant differences in their perception of the therapeutic relationship (real relationship, therapeutic alliance) in phone versus video sessions. Therapists reported higher levels of professional self-doubt in video sessions compared to phone sessions.

The results regarding the UTAUT subscales revealed that therapists perceived the use of video as significantly less easy compared to the use of phone and, in the meantime, they also reported greater external pressure but also more professional support for the use of video. Intention to use video or phone for sessions in the future did not differ significantly, see Table 4.

### 3.4. Moderators of the Difference between Phone and Video Sessions

The ANOVAs assessing the potential moderating roles of therapists’ years of clinical experience and number of present phone/video sessions were nonsignificant for all the dependent variables (attitudes towards teletherapy technology, working alliance, real relationship, and professional self-doubt).

Previous experience with providing teletherapy via phone or video conferencing was not significant to moderate the difference between therapists’ attitudes towards teletherapy via phone versus videoconferencing. However, the two-way interaction between previous phone experience x media (phone/video) significantly predicted attitudes F(1,114) = 6.17, *p* = .014, *η*^2^ = 0.01. The remaining two-way interactions (phone experience x video experience, and video experience x media (phone/video) and the three-way interaction (phone experience x video experience x media (phone/video)) did not reach significance. Post hoc pairwise comparisons showed that therapists with previous phone therapy experience had significantly more positive overall attitudes towards phone therapy (M = 3.37, SE = 0.06) compared to those without such experience (M = 3.01, SE = 0.10), *t*(113) = 3.135, *p* = .010, *η*^2^ = 0.01.

**Professional Self-doubt.** The three-way mixed ANOVA conducted to assess differences between professional self-doubt in phone versus video sessions showed significant main effect for media (phone/video) and for previous experience with phone and with video on professional self-doubt, F(2,114) = 488.47, *p* <.01, *η*^2^ = .33. The two-way interaction for video experience x media (phone/video) was also significant, F(2,114) = 11.741, *p* < 0.01, *η*^2^ = .05. The remaining two-way interactions and the three-way interactions were not significant (all *p* > .05). Post hoc pairwise comparisons showed that therapists with previous phone experience reported less professional self-doubt in their phone practice (M = 1.05, SE = 0.08) compared to those with no previous experience (M = 1.84, SE = 0.15), *t*(113) = −4.69, *p* < .001, *η*^2^ = 0.01.

**Therapeutic Relationship**. Three-way ANOVA also showed a significant main effect of previous phone experience on the working alliance F(2,114) = 10.07, *p* = .02, *η*^2^ = 0.08. That is, therapists with prior phone experience reported stronger working alliance (M = 4.04, SE = 0.05) compared to those with no phone experience (M = 3.60, SE = 0.08), *t*(113) = −3.87, *η*^2^ = 0.01. Three-way ANOVA also revealed a significant main effect of previous phone experience on real relationship scores, F(2,114) = 9.18, *p* < .01, *η*^2^ = 0.06, with therapists with prior phone experience reporting a better perceived real relationship with patients (M = 3.84, SE = 0.04) than those without (M = 3.58, SE = 0.06, *t*(113) = −2.82, *η^2^* = 0.01. The remaining two-way and the three-way interactions were not significant (*p* < .05).

## 4. Discussion

The pandemic provided a unique time for therapists, where they gained *en masse* experience with teletherapy. Now, post-pandemic, teletherapy has remained a popular therapy format. Despite the vast empirical literature on therapists’ attitudes towards teletherapy, little research has distinguished their perspectives on sessions via phone and sessions via videoconferencing. In this study we aimed to examine therapists’ perceptions of teletherapy sessions via the phone versus via sessions via videoconferencing. More specifically, we assessed differences in therapists’ attitudes toward teletherapy technology, their levels of professional self-doubt, and perceptions of the therapeutic relationship in phone versus video sessions. We also considered the influence of therapists’ level of clinical experience, previous experience with conducting sessions via phone and video, and number of sessions provided during the time of study on the identified differences.

We found no differences in therapists’ attitudes toward conducting therapy via phone and video or in their perceived quality of therapeutic relationships in phone versus video sessions. However, therapists reported significantly more professional self-doubt in sessions conducted via videoconferencing than in sessions conducted via phone. Furthermore, therapists with more years of clinical experience reported less professional self-doubt, and more positive attitudes in both phone and video sessions. Therapists who already had previous experience with providing teletherapy via the phone perceived the therapeutic relationship as significantly stronger in their phone sessions than in their video sessions both in terms of working alliance and real relationship.

Thus, the surveyed therapists who conducted sessions via phone and video had similar attitudes towards phone and video technology and also had similar experiences regarding the therapeutic relationship in phone sessions rather than in video sessions. These experiences were further boosted by more years of clinical experience and previous experience with conducting sessions via the phone. Although there are no other empirical studies that examined therapists’ perspectives on teletherapy via phone versus videoconferencing, our results are in line with studies that compared therapists’ perspectives on teletherapy versus in-person therapies. Specifically, previous studies reported that therapists’ perceptions of the therapeutic alliance and the real relationship were similar in teletherapy via videoconferencing versus in in-person [12].

The similar attitudes towards phone and video sessions, as well as similar ratings in perceived real relationship and working alliance, suggest that the type of teletherapy format does not constrain the quality of the therapeutic relationship per se [11,50,51,52,53]. The therapeutic process might depend more on the therapists’ level of experience than the teletherapy technology that was used.

These results fit with previous studies on teletherapy more broadly, in that familiarity with the teletherapy technology plays an important role in shaping therapists’ attitudes and experiences with teletherapy [8,17,25,26], as well as their level of professional self-doubt [54].

It might not be surprising that professional self-doubt was higher in teletherapy sessions via videoconferencing than in phone sessions. In previous studies conducted during the pandemic that compared teletherapy to in-person therapies, therapists reported higher levels of professional self-doubt in teletherapy via videoconferencing than in in-person sessions. During the pandemic, the therapists probably gained many years of experience talking on the phone in various contexts. In contrast, video conferencing technology was relatively new for many therapists. With more practice and experience in using the teletherapy technology, therapists can gain more confidence. This practice and training ultimately give therapists more freedom to choose the teletherapy technology that best suits their patient’s needs [55].

Within attitudes towards phone and video therapy, the comparison of UTAUT-T subscales showed that despite therapists reporting that phone therapy was perceived as easier to use and that there was a greater external pressure and professional support for the use of video, at least during the time of early pandemic, when our study was conducted. The increased institutional and professional push to use video reported in this study is in line with the pre-pandemic reviews that have noted the increasing use of video over other technology, such as phone, for remote mental care [26]. Post-pandemic studies show the widespread use of video for teletherapy, which clearly demonstrated the impact of external pressure, in this case, in the form of not only institutional pressure but also public health necessity, on adopters’ acceptance of technology [56,57]. Furthermore, the study’s findings align with previous research [16,26], at least about the initial technical challenges of practicing via videoconferencing [26].

### Limitations

Several limitations of this study may be highlighted. First, the findings from our small subsample might not generalize to teletherapists more broadly. Among the total sample of 1200 respondents, only 115 reported practicing therapy via videoconferencing and phone. The generalizability of the findings was further limited by selection bias. Participation in the study was voluntary, with psychotherapists being recruited via professional email listservs, social media, and individual contacts across the United States, Canada, China, and Europe. Those who chose to participate might inherently differ from those who did not [57]. Second, the nuance around therapists’ experience with the teletherapy technology might not have been captured by the questions we asked about previous experience with phone or video sessions (yes/no, number of sessions). A more detailed evaluation of therapists’ exposure and experience with these teletherapy technologies in clinical training, supervision, or personal contexts could provide a more nuanced understanding of the role of these factors in technology acceptance. Similarly, it would be relevant to assess the service context of the surveyed therapists. The extent to which the therapists experienced a top–down organizational directive to adopt one teletherapy format over the other might have influenced their responses [31].

## 5. Conclusions

Therapists’ attitudes and perceptions of the therapeutic relationship are generally similar for teletherapy via phone and video. However, therapists may experience higher levels of professional self-doubt in sessions conducted via videoconferencing than in sessions conducted via the phone. Prior experience with conducted teletherapy via the phone resulted in more positive attitudes towards phone sessions. This suggest that familiarity with videoconferencing technology might be more important in shaping therapists’ attitudes and confidence than the type of technology per se. The study findings highlight the adaptability of therapists in the face of new technologies.

## Figures and Tables

**Table 1:** Therapists’ Characteristics (*n* = 117).

	*n*	%
Ethnicity		
White European, European American	90	76.9
Asian or Asian Indian	14	12.0
Hispanic, Latinx, Spanish	4	3.4
Middle Eastern	3	2.6
Black or African American	1	0.9
First Nations	1	0.9
Other	4	3.4
Profession		
Psychologist	59	50.4
Social worker	12	10.3
Counsellor	16	13.7
Medical doctor	18	15.4
Psychoanalyst	11	9.4
Marriage and family therapist	4	3.4
Other	20	17.1
Licensure status		
Licensed	110	94.0
Trainee	7	6.0
Clinical experience in years		
0–4	10	8.6
5–8	15	12.8
9–12	8	6.8
13–16	9	7.7
17 or more	75	64.1
Work setting		
Private practice	98	83.8
Outpatient clinic	23	19.7
Hospital	10	8.6
Inpatient clinic	2	1.7
Over the phone/online only	1	0.9
Other	9	7.8
Theoretical orientation *		
CBT	33	28.2
Humanistic	25	21.4
Psychodynamic	76	65.0
Psychoanalytic	75	64.1
Integrative	48	41.0
Systemic	15	12.8
Other	10	0.1
Previous experience of providing therapy *by phone*		
Yes	88	75.2
No	29	24.8
Previous experience of providing therapy *by video*		
Yes	62	53.0
No	55	47.0
Number of sessions per week *by phone* previously		
0	61	52.1
1	23	19.7
2	15	12.8
3	8	6.8
>3	10	8.6
Number of sessions per week *by video* previously		
0	79	67.5
1	19	16.2
2	5	4.3
3	5	4.3
>3	9	8.1

Note: * Multiple responses were possible; Categories are ordered by frequency.

**Table 2:** Zero-Order Correlations Between Therapists’ Previous And Present Experience And The Study Variables (*n* = 117).

	Age	Clinical Experience			Number of Patients Now	
		General	Phone	Video	Phone	Video
Video						
UTAUT-T	.16	.09	.14	.26 **	−.16	.21 *
PSD	−.40 ***	−.34 ***	−.34 ***	−.26 **	.06	−.10
WAI	.04	−.01	.25 **	.00	−.01	.18 *
RRI	−.03	.09	.22 *	.05	−.16	.17 *
Phone						
UTAUT-T	.31 ***	.28**	.27 **	.21 *	−.13	−.01
PSD	−.45 ***	−.39 ***	−.40 ***	−.29 **	.05	−.20 *
WAI	.11	.16	.32 ***	.15	.07	.10
RRI	.09	.15	.26 **	.05	−.02	.08

Note. * p < .05. ** *p* < .01. *** *p* < .001. UTAUT-T = Unified Theory of Acceptance and Use of Technology–Therapist Scale; PSD = Professional Self-Doubt; RRI = Real Relationship Inventory; WAI = Working Alliance Inventory–Short Revised–Therapist.

**Table 3:** Zero-order correlations between the standardized scales for phone and video therapy (*n* = 115).

	Video				Phone			
	UTAUT-T	PSD	WAI	RRI	UTAUT-T	PSD	WAI	RRI
**Video**								
UTAUT-T	--	−.33 ***	.34 ***	.28 **	.67 ***	−.26 **	.31 ***	.18
PSD		--	−.13	−.23 *	−.39 ***	.78 ***	−.15	−.22 *
WAI			--	.58 ***	.21 *	−.13	.79 ***	.48 ***
RRI				--	.29 **	−.17	.48 ***	.64 ***
**Phone**								
UTAUT-T					--	−.43 ***	.30 ***	.36 ***
PSD						--	−.25 **	−.35 ***
WAI							--	.61 ***
RRI							.	--

Note. * p < .05. ** *p* < .01. *** *p* < .001. UTAUT-T = Unified Theory of Acceptance and Use of Technology–Therapist Scale; PSD = Professional Self-Doubt; RRI = Real Relationship Inventory; WAI = Working Alliance Inventory–Short Revised–Therapist.

**Table 4:** Comparison of therapists’ attitudes towards teletherapy technology, professional self doubt, the real relationship, and working alliance in phone and video sessions (*n* = 11.7).

Measure	Video		Phone		*t*		
	*M (SD)*	*Range*	*M (SD)*	*Range*		*p*	*d*
UTAUT-T							
Mean	3.24 (.50)	1.84–4.63	3.29 (.58)	1.63–4.89	1.16	.248	.087
Therapy quality	3.05 (.59)	1.77–4.56	3.09 (.78)	1.11–5.00	0.70	.486	.057
Ease of use	3.60 (.76)	1.25–5.00	3.99 (.71)	2.00–5.00	6.52	<.001	.538
Pressure	2.91 (.98)	1.00–5.00	2.67 (.97)	1.00–5.00	−3.45	<.001	.246
Support	3.77 (.73)	1.00–5.00	3.42 (.89)	1.00–5.00	−5.04	<.001	.432
Convenience	3.17 (1.01)	1.00–5.00	3.24 (.98)	1.00–5.00	1.24	.218	.073
Intention	3.24 (1.15)	1.00–5.00	3.13 (1.10)	1.00–5.00	−1.34	.184	107
PSD	2.40 (.82)	1.00–4.44	1.23 (.87)	0.00–3.56	−22.57	<.001	1.386
RRI	3.80 (.45)	2.63–4.71	3.75 (.51)	2.67–4.88	−1.24	.216	.098
WAI	3.96 (.67)	1.00–5.00	3.92 (.67)	1.00–5.00	−0.66	.509	.040

Note. UTAUT-T = Unified Theory of Acceptance and Use of Technology–Therapist Scale; Therapy Quality = UTAUT-T Therapy Quality Expectation. PSD = Professional Self-Doubt; RRI = Real Relationship Inventory; WAI = Working Alliance Inventory–Short Revised–Therapist.

## Data Availability

Data is available from the authors by reasonable request.
